# Combined Transcriptome and Proteome Analysis of Anthers of AL-type Cytoplasmic Male Sterile Line and Its Maintainer Line Reveals New Insights into Mechanism of Male Sterility in Common Wheat

**DOI:** 10.3389/fgene.2021.762332

**Published:** 2021-12-17

**Authors:** Miaomiao Hao, Wenlong Yang, Tingdong Li, Muhammad Shoaib, Jiazhu Sun, Dongcheng Liu, Xin Li, Yingbin Nie, Xiaoming Tian, Aimin Zhang

**Affiliations:** ^1^ State Key Laboratory of Plant Cell and Chromosome Engineering, Institute of Genetics and Developmental Biology/Innovative Academy of Seed Design, Chinese Academy of Sciences, Beijing, China; ^2^ University of Chinese Academy of Sciences, Beijing, China; ^3^ Institute of Vegetables and Flowers, Chinese Academy of Agricultural Sciences, Beijing, China; ^4^ Institute of Crop Research, Xinjiang Academy of Agri-Reclamation Sciences, Shihezi, China

**Keywords:** AL-type cytoplasmic male sterility, AL18A, transport, anthers development genes, wheat

## Abstract

Cytoplasmic male sterility (CMS) plays an essential role in hybrid seeds production. In wheat, *orf279* was reported as a CMS gene of AL-type male sterile line (AL18A), but its sterility mechanism is still unclear. Therefore, transcriptomic and proteomic analyses of the anthers of AL18A and its maintainer line (AL18B) were performed to interpret the sterility mechanism. Results showed that the electron transport chain and ROS scavenging enzyme expression levels changed in the early stages of the anther development. Biological processes, i.e., fatty acid synthesis, lipid transport, and polysaccharide metabolism, were abnormal, resulting in pollen abortion in AL18A. In addition, we identified several critical regulatory genes related to anther development through combined analysis of transcriptome and proteome. Most of the genes were enzymes or transcription factors, and 63 were partially homologous to the reported genic male sterile (GMS) genes. This study provides a new perspective of the sterility mechanism of AL18A and lays a foundation to study the functional genes of anther development.

## Introduction

Heterosis refers to the phenomenon that offspring are superior to the parents in terms of viability, stress resistance, growth potential, and other traits (Chen and Liu, 2014). Heterosis can be utilized to develop new varieties with high yield, good quality, and stress resistance. However, it is challenging to utilize heterosis in self-pollinated crops. Therefore making it necessary to develop male sterile lines (Berlan, 2018). The cytoplasmic male sterile (CMS) line is one of the leading male sterility systems used to produce hybrid seeds. The CMS genes exist in mitochondria while the restorer genes (RF) exist in the nucleus. The CMS/RF system is produced due to abnormal between the nucleus and the cytoplasm (Tang et al., 2017). Different types of CMS lines have been reported in wheat, e.g., T-CMS, K-CMS, V-CMS, AL-CMS, etc. (Gupta et al., 2019). Studies on the male sterility mechanism of wheat can accelerate the utilization of heterosis in wheat.

The molecular mechanisms of cytoplasmic male sterility can be classified into four types: cytotoxicity model, energy deficit model, abnormal PCD model, and retrograde regulation model (Chen and Liu, 2014). In the cytotoxicity model, CMS genes encode toxic proteins, i.e., URF13, ORF138, or ORF79, inhibiting cell growth and development (Korth and Levings, 1993; Duroc et al., 2005; Wang et al., 2006). In the energy deficit model, CMS genes primarily encode transmembrane proteins in the inner membrane of mitochondria and the chimeric part of the mitochondrial genes, affecting the electron transport chain and ATP production leading to energy deficiency and pollen abortion (Sabar et al., 2003; Logan, 2006; Duroc et al., 2009). Timely PCD in the tapetum is crucial to pollen development (Zhang et al., 2016; Yu and Zhang, 2019). In abnormal PCD models, tapetum cells advance or delay PCD, leading to abnormal nutrient absorption of microspores and pollen abortion, like *WA352* (Reape and McCabe, 2010; Falasca et al., 2013; Luo et al., 2013). In the retrograde regulation model, CMS genes in mitochondria regulate nuclear gene expression through various retrograde signals, thereby regulating pollen fertility, like *orf355* (Xiao et al., 2020). Wan *et al.* classified the reported genic male sterility genes into four categories: transcriptional regulation, lipid metabolism, polysaccharide metabolism, and others (Wan et al., 2019). The main component of pollen exine, sporopollenin, is synthesized by lipid and phenylpropane metabolism in the tapetum cells (Zhang et al., 2016; Wan et al., 2020).

At present, many genes related to anthers development have been reported in rice and *Arabidopsis* (Wan et al., 2019). Nevertheless, the mechanism of wheat anther development is still unknown. The advancement in biotechnology, transcriptome, proteome, metabolome, and other omics methods has collectively been utilized in anthers development studies (Zheng et al., 2014; Xing et al., 2018; Cheng et al., 2019; Ning et al., 2019; Zhang et al., 2021), accelerated the research of anther development. The transcriptome analysis of the *Aegilops kotschyi* thermo-sensitive cytoplasmic male sterile and fertile lines showed that genes related to phenylpropane biosynthesis and jasmonic acid biosynthesis pathway, regulated by MYB transcription factors, were up-regulated in fertile anthers during fertility conversion (Ye et al., 2017). Yang et al. (2018) compared the transcriptome of the sterile line (EP26A) and the maintainer line (EP26) and identified 1716 DEGs (differentially expressed genes) at different anther development stages. GO (Gene Ontology), and KEGG (Kyoto Encyclopedia of Genes and Genomes) analysis showed that these genes were mainly involved in redox, carbohydrate, and amino acid metabolism. Proteomic analysis of the anthers of the Yunnan early rice A (ZD-CMS) and its maintainer line revealed that the down-regulation of the 45 DEPs (differentially expressed proteins) involved in the carbohydrate metabolism and stress response in male sterile line. The ROS content in the anther mitochondria of ZD-CMS was increased, and the ultrastructural analysis revealed the destruction of mitochondria (Yan et al., 2014). Proteome and transcriptome analysis of flower buds from the sterile line, Shanan2A and its maintainer Shanan2B, of *Brassica napus* revealed the involvement of DEGs in carbon metabolism, lipid and flavonoid metabolism, mitochondrial electron transport and ATP synthesis pathways. The DEPs are mainly related to carbohydrate metabolism, energy metabolism, and genetic information processing pathways (Ning et al., 2019). In wheat AL-CMS type sterile line (AL18A), *orf279* was reported as the CMS gene (Hao et al., 2021; Melonek et al., 2021), but the sterility mechanism needs further study.

In this study, transcriptomics and proteomics were used to analyze variations between AL18A and AL18B at different stages of anthers development, thus explored the biological processes and metabolic pathways related to the AL18A pollen abortion and identified the genes related to anthers development. This study laid a foundation for the sterility mechanism analysis of wheat AL18A cytoplasmic male sterile line.

## Materials and Methods

### Plant Material

AL18A, a stable wheat AL-CMS line, was developed from a cross between AL-CMS line 781A and Xindong18 (recurrent parent). The maintainer line Xindong18 will be mentioned as AL18B, hereafter. The plant material was obtained from the Xinjiang Academy of Agri-Reclamation Sciences and planted in the Institute of Genetics and Developmental Biology experimental farm (2018). Anthers of AL18A and AL18B collected at the tetrad, uni-nucleate, bi-nucleate, and tri-nucleate stages were immediately frozen in liquid nitrogen and stored at −80°C, The developmental stage of pollen was determined using the procedure described byBrowne et al. (2018).

### Transcriptomic Sequencing

Samples for RNA-seq analysis were prepared from the wheat lines AL18A and AL18B at four different stages of anther development with three biological replications, which made 2×4×3 = 24 libraries in total. Total RNA was extracted using RNEasy Plant Mini Kit (Qiangen, Germany). RNA quality was evaluated on 1% agarose gel, and the purity was checked on a Nano-Photometer spectrophotometer (IMPLEN, CA, United States). RNA concentration was measured using the Qubit RNA Assay Kit and a Qubit 2.0 Fluorometer (Life Technologies, CA, United States), and RNA integrity was assessed using the RNA Nano 6000 Assay Kit on a Bioanalyzer 2100 system (Agilent Technologies, CA, United States). RNA quality testing is done by the cooperative company (BGI, China). The total amount of RNA in each sample was greater than 4 μg, OD260/OD280 ranged from 1.8 to 2.2, OD260/OD230 ranged from 2 to 2.4, and RIN value was greater than 6.3. Ribosomal RNA was depleted using the EpicentreRibo-Zero Gold Kit (Illumina, San Diego), and after purification, depleted rRNA was fragmented into small pieces using fragmentation buffer. The fragmented RNA was reverse transcribed to create the cDNA libraries according to the mRNA-Seq sample preparation kit (Illumina), and paired-end sequencing (2 × 150 bp) was performed on Illumina Hiseq2500 sequencer at BGI company. Trimmomatic (V0.32) (Bolger et al., 2014) was used for data quality filtering, and the bases with Phred quality score mass above 20 were retained. After removing the smaller reads (<25) and one-end reads, the filtered data were aligned to the Chinese Spring wheat reference genome (IWGSC RefSeq V1.0) by HISAT2 (Trapnell et al., 2009). The htseq-count was used to estimate the expression value of each transcript, and the differentially expressed genes (DEGs) were analyzed with DEGseq software (Robinson et al., 2010). P-value < 0.05 and |log2 (fold change) | *>*1 were used as the threshold values for significantly different expression levels.

### DIA Quantification Proteomics

Samples for DIA analysis were prepared from the wheat lines AL18A and AL18B at two different stages (uni-nuclear stage and bi-nuclear stage) of anther development with three biological replications. Each sample was collected with more than 100 mg anther for protein extraction (Imin et al., 2001).The protein samples were sequentially digested with trypsin in a 1:40 ratio for 12 h (4 h + 8 h) at 37°C. Enzymatic peptides were desalted using a Strata X column and vacuumed to dryness. The trypsin digestion was analyzed by LC-MS/MS technique using an Ultimate 3000 nanoLCsystem (Thermo Scientific) coupled to an Orbitrap Fusion Lumos mass spectrometer (Thermo Scientific) operated in either DDA (Data-dependent Acquisition) or DIA (Data-independent Acquisition) acquisition mode at a gradient time of 180 min. Andromeda search engine within MaxQuant (Cox and Mann, 2008) was used for DDA data identification, and the results were used for spectral library construction.

Reference protein database (https://urgi.versailles.inra.fr/download/iwgsc/IWGSC RefSeq_Annotations/v1.0/). For large-scale DIA data, mProphet algorithm was used to complete analytical quality control, thus obtaining a large number of reliable quantitative results. MSstats (Choi et al., 2014) was used to evaluate significant differences in proteins or peptides from different samples. Differentially expressed proteins, having a fold change difference of >2 at the adjusted P-value of <0.05, were considered significant.

### Bioinformatics Analysis

Genes identified through the transcriptomics and proteomics were annotated using the following databases: NCBI non-redundant protein sequences (Nr); Kyoto Encyclopedia of Genes and Genomes (KEGG); Gene Ontology (GO); Pfam (a database of protein families); Swiss-Prot (a manually annotated and reviewed protein sequence database); and Eukaryotic orthologous groups (KOGs). Bingo (V2.44) was used for GO enrichment analysis, GO terms with P-value < 0.05 were defined as significantly enriched in DEGs/DEPs, and KEGG functional enrichment analysis of the DEGs/DEPs was executed by KOBAS software. Protein-protein interaction (PPI) was observed using the STRING database (http://string-db.org). Bi-direction clustering of genes and samples was done in the R package.

## Results

### Alterations in the Electron Transport Chain and ROS Level Can Affect Pollen Fertility of AL18A

**FIGURE 1:**
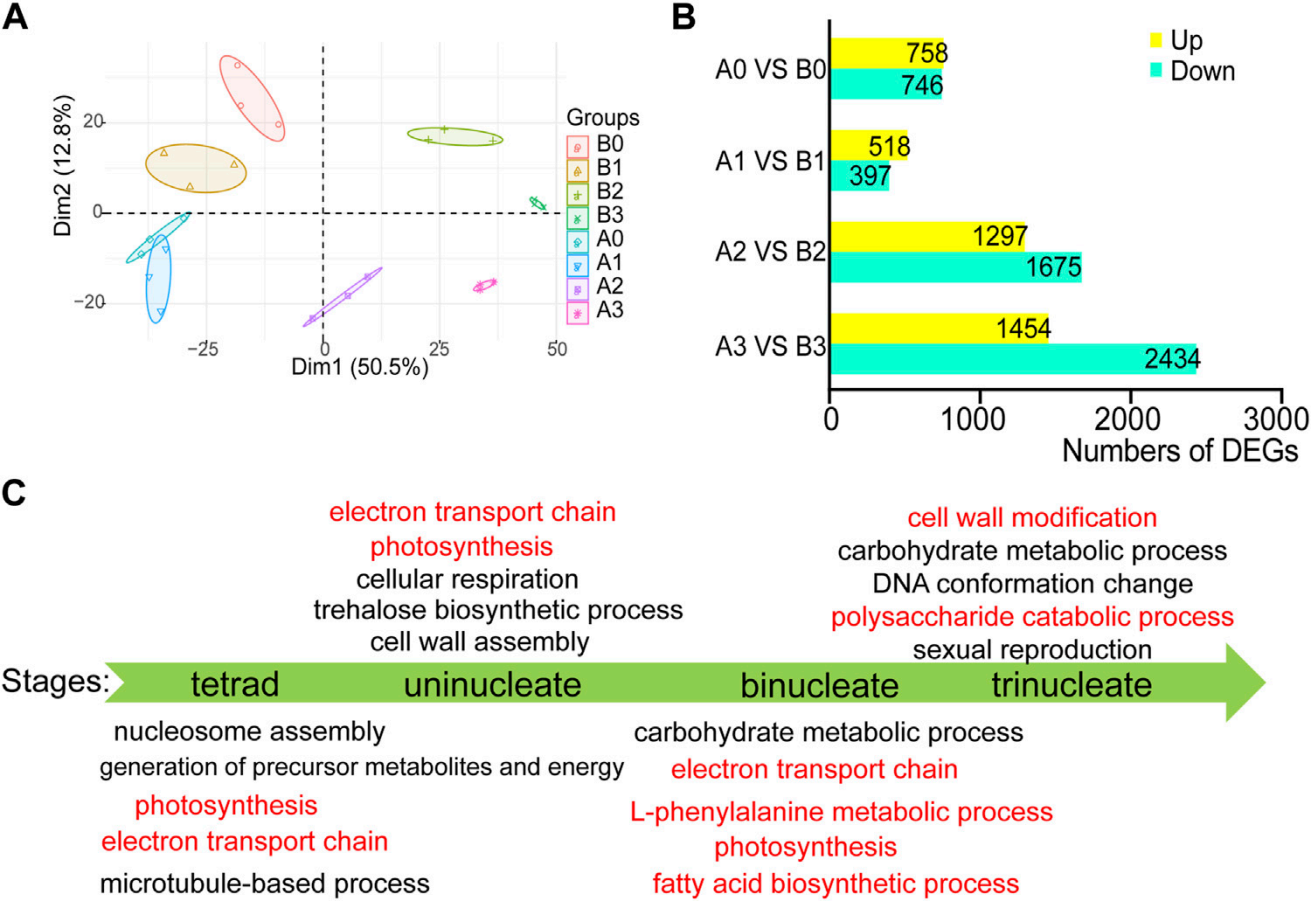
Differentially expressed genes and GO function analysis in AL18A at different developmental stages. **(A)** Principal component analysis (PCA) of RNA-seq data for four stages of wheat anther development. B0, B1, B2, and B3 represent the tetrad, uni-nucleate, bi-nucleate and tri-nucleate stages of anther development of AL18B; A0, A1, A2, and A3 represent the tetrad, uni-nucleate, bi-nucleate and tri-nucleate stages of anther development of AL18A, respectively. **(B)** The number of DEGs between AL18A and AL18B at different anther developmental stages. DEGs: differentially expressed genes. **(C)** GO function enrichment analysis of DEGs between AL18A and AL18B at different anther development stages.

More than 12 GB of high-quality RNA-seq data were generated from each sample using the Illumina sequencing platform. The reads were mapped to IWGSCWGA v1.0 with a unique mapped ratio of 69.43–83.32% (Supplementary Table S1). To gain insight into the transcriptome dynamic of anthers development, we performed principal component analysis for the four-time series of AL18A and AL18B (Figure 1A). The plant background and timing of tetrad, uni-nuclear, bi-nuclear, and tri-nuclear stages for anther development are in line. 1504, 916, 2973, and 3889 DEGs between the AL18A and AL18B were obtained at the tetrad, uni-nuclear, bi-nuclear, and the tri-nuclear stages of anther development, respectively (Figure 1B). The number of DEGs gradually increased from the uni-nuclear stage to the tri-nuclear stage, this result is consistent with our previous discovery that AL18A anthers have delayed PCD in the tapetal layer at the uni-nuclear stage (Hao et al., 2021). It is suggested that the early abnormal development of the anther of AL18A was near the uni-nuclear stage. In addition, 153 DEGs were present at all four developmental stages (Supplementary Figure S1; Supplementary Table S2).

To study the molecular mechanism of the pollen abortion in the AL18A line, GO enrichment analysis was performed on the DEGs at four stages of anther development (Figure 1C; Supplementary Table S3). Results showed that the L-phenylalanine metabolic process, fatty acid biosynthetic process, cell wall modification, and the polysaccharide catabolic process were abnormal in the late anther development of AL18A. Fatty acid biosynthetic process and L-phenylalanine metabolic process are known to be involved in pollen wall development (Wan et al., 2019). These DEGs involved in biological processes are consistent with previous reports and provide strong evidence for the pollen abortion of AL18A. In addition, many DEGs participate in electron transport chains and photosynthesis during the early anther development of AL18A. This result is consistent with the genetic background of AL18A and AL18B, because they have the same nucleus but different cytoplasm. Combined with our previous studies that *orf279* is the CMS gene of AL18A, the first 96 amino acids of ORF279 and ATP8 were identical (Hao et al., 2021), therefore, *orf279* may lead to abortion of AL18A by affecting the electron transport chain.

**FIGURE 2:**
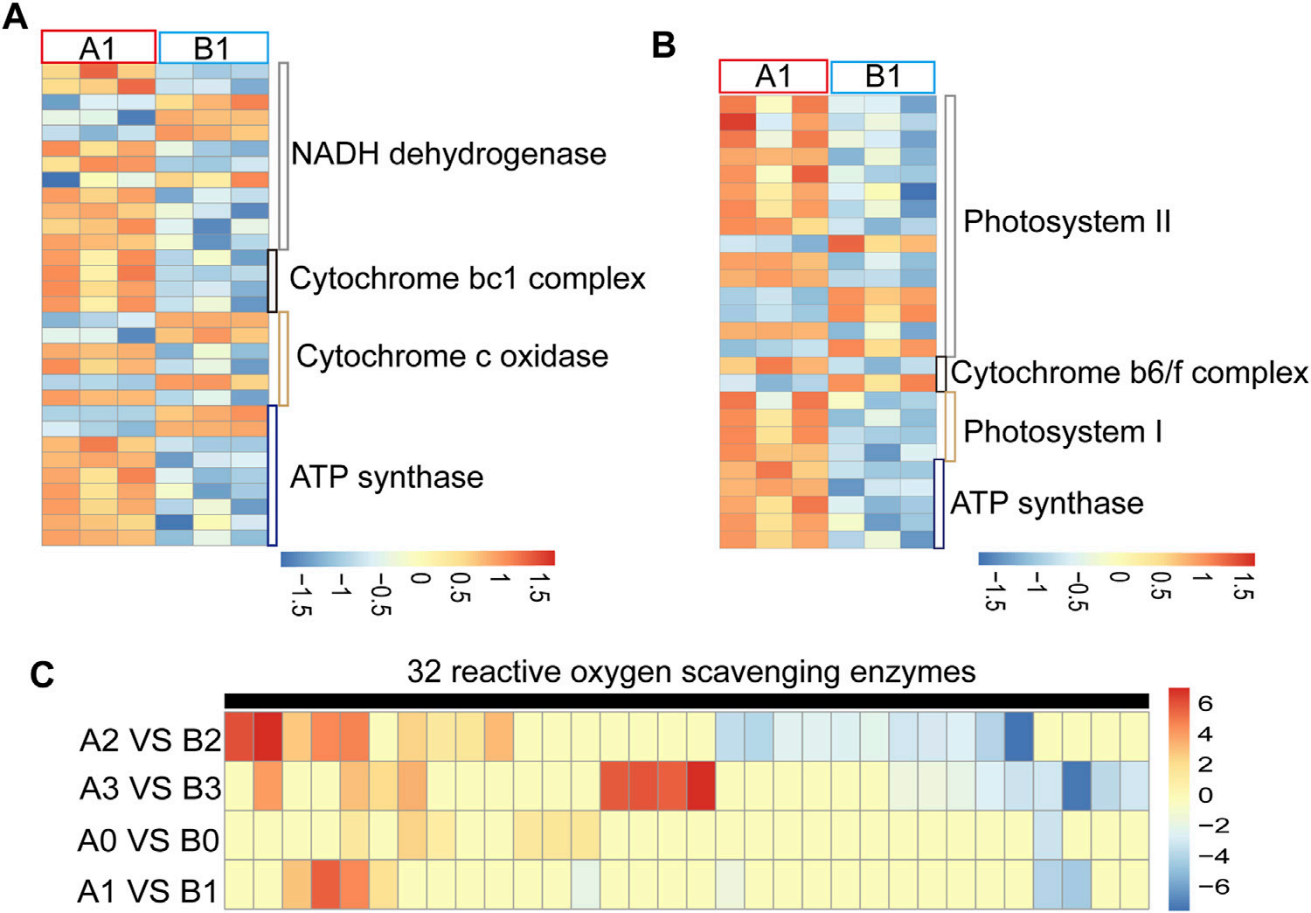
Expression analysis of DEGs involved in electron transport chain and ROS scavenging enzymes. **(A)** Expression analysis of DEGs in mitochondrial electron transport chains of AL18A and AL18B in uni-nucleate stage. **(B)** Expression analysis of DEGs in chloroplasts electron transport chains of AL18A and AL18B in uni-nucleate stage. **(C)** Expression analysis of DEGs in ROS scavenging enzymes of AL18A and AL18B in different anther development stages.

Most of the CMS genes interfere with the mitochondrial electron transport chain, resulting in energy deficiency or ROS level abnormality leading to advance or delay of tapetal PCD and eventually cause pollen abortion (Chen and Liu, 2014). We found that 31 of the oxidative phosphorylation-related genes were abnormally regulated at the uni-nuclear stage in AL18A anthers (Figure 2A; Supplementary Table S4) and classified into four clusters. Cluster one included 12 NADH dehydrogenase-related genes, cluster two included four cytochrome bc1 complex related genes, cluster three included six cytochrome c complex related genes, and cluster four included nine ATP synthases. In addition, most of the 26 photosynthesis related genes were up-regulated at uni-nuclear stage in AL18A anthers (Figure 2B; Supplementary Table S5). These genes are associated with photosystem I, photosystem II, cytochrome b6f complex, and ATP synthase. We can see that these DEGs are mainly involved in the electron transport chain. Studies have shown that electron transport chain abnormalities can affect ROS levels (Van Aken and Van Breusegem, 2015). We detected that the ROS scavenging enzyme expression changes at different stages of anther development in AL18A and AL18B (Figure 2C; Supplementary Table S6). 32 ROS scavenging enzyme related genes were abnormally regulated in AL18A at anther development stage, and 30 of them encode peroxidases. 7, 8, 20, and 17 ROS scavenging enzymes were differentially expressed at the tetrad, uni-nuclear, bi-nuclear, and tri-nuclear stages. We found that six of the peroxidases were up-regulated, and one was down-regulated in the tetrad stage (Supplementary Table S6). Previous studies have shown that during anther development, the process of PCD in tapetum is regulated by ROS levels (Yu and Zhang, 2019). Differential expression in the ROS scavenging enzymes suggests changes in ROS levels in the AL18A, this is associated with our previous discovery of delayed PCD in the tapetal cells of AL18A (Hao et al., 2021).

### Proteomic Analysis of AL18A and AL18B

**FIGURE 3:**
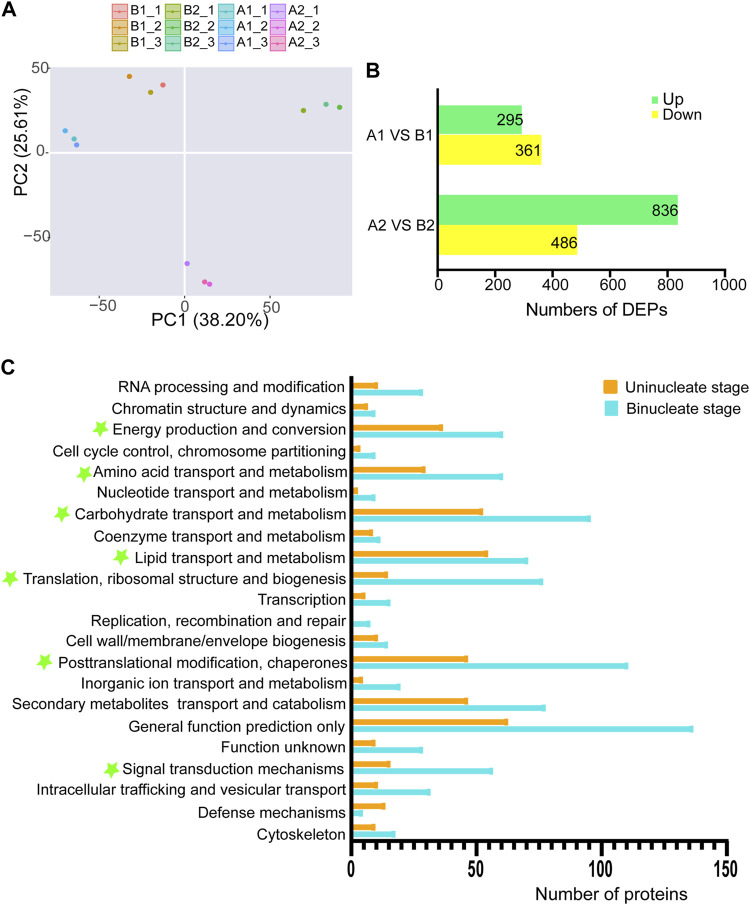
Differentially expressed proteins (DEPs) and functional classification of DEPs from anther proteome between AL18A and AL18B. **(A)** Principal component analysis of proteomic data for two stages of wheat anther development. **(B)** The number of DEPs between AL18A and AL18B at different anther developmental stages. **(C)** Functional classification of DEPs at two stages of anther development. All proteins aligned with the KOG database were divided into 24 clusters.

To better understand the sterility mechanisms underlying AL18A, we performed the proteomic analysis of AL18A and AL18B. Principal component analysis showed that the proteomic dynamic of anthers development is in line with the plant background and the timing of uni-nuclear and bi-nuclear stages (Figure 3A). The number of DEPs between AL18A and AL18B gradually increased from the uni-nuclear stage (656) to the bi-nuclear stage (1322) (Figure 3B), and these results were consistent with the transcriptome analysis. Eukaryotic orthologous groups (KOGs) analysis was conducted to understand the potential functions of the DEPs globally (Figure 3C). At the uni-nuclear and bi-nuclear stages, most of the DEPs were categorised into post-translational modification and chaperones; translation, ribosomal structure and biogenesis; carbohydrate transport and metabolism; amino acid transport and metabolism; energy production and conversion; lipid transport and metabolism; and signal transduction mechanisms, suggested that the male sterility in AL18A might be related to the disruption of anther cells metabolism at multiple levels.

**FIGURE 4:**
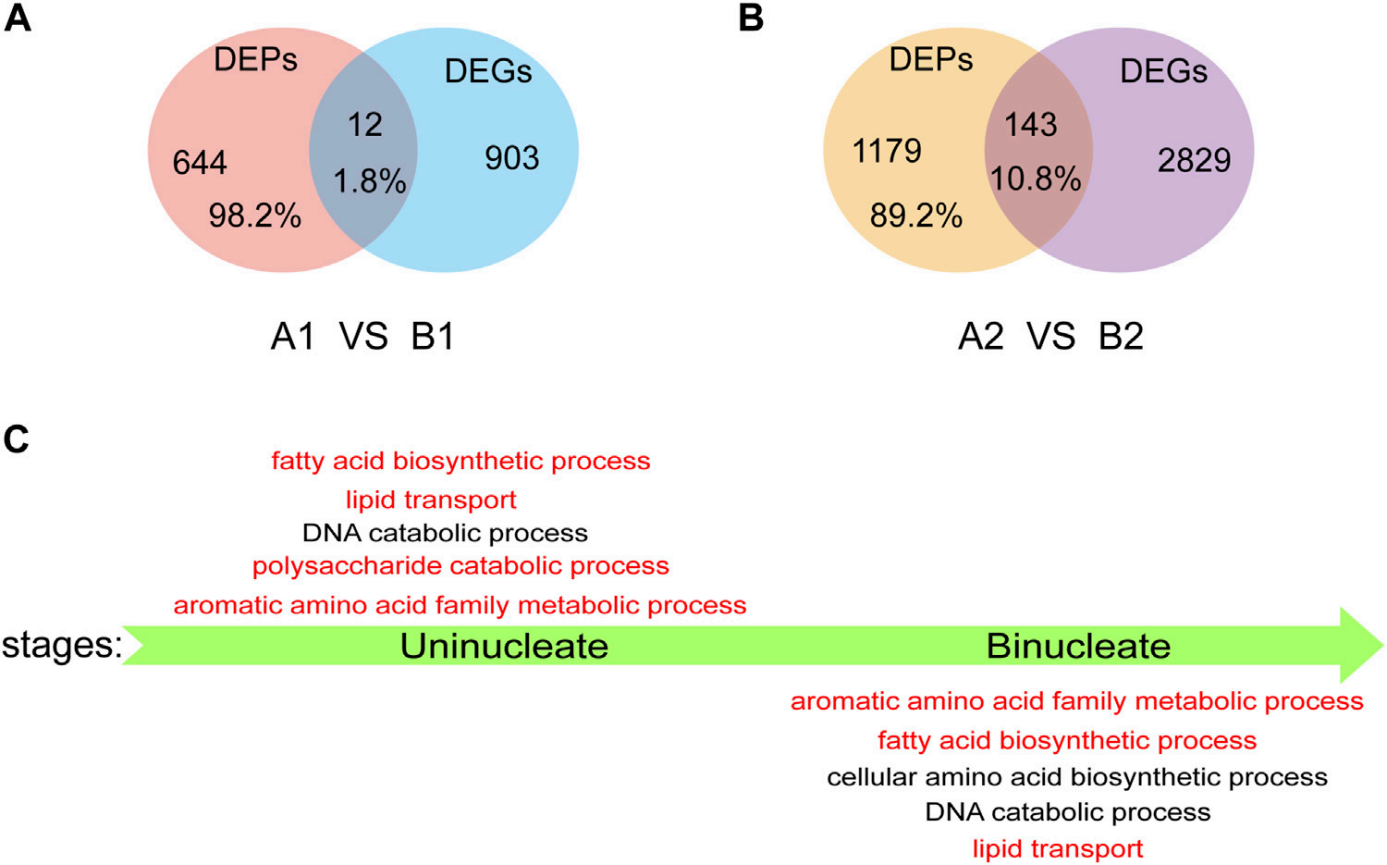
Analysis of DEPs detected only by proteomics. **(A)** Venn diagrams of DEPs and DEGs in uni-nucleate stage. **(B)** Venn diagrams of DEPs and DEGs in bi-nucleate stage. **(C)** GO function enrichment analysis of DEPs detected only by proteome in uni-nucleate and bi-nucleate stages.

We performed a conjoint analysis in AL18A and AL18B to explore the correlation between the DEGs and DEPs. At the uni-nuclear and bi-nuclear stages, only 1.8% (12/656) and 10.8% (143/1322) of the DEPs overlapped with the DEGs, a total of 146 genes were differentially expressed at both RNA and protein levels (Figures 4A,B; Supplementary Table S7), respectively, indicating a poor correlation between the DEPs and DEGs species accumulation patterns as described in previous studies (Han et al., 2018). These results also infer that proteomics can generate information different from transcriptomics. Therefore, GO analysis was performed on DEPs identified only by the proteome in the uni-nuclear and bi-nuclear stages (Figure 4C; Supplementary Table S8). The result indicated that at uni-nuclear and bi-nuclear stages, the DEPs were involved in fatty acid biosynthesis, lipid transport, aromatic amino acid family metabolism, and DNA catabolism. In addition, polysaccharide catabolic process was also abnormal in uni-nuclear stage of AL18A. Previous studies have shown that abnormal lipid and polysaccharide metabolism often leads to male sterility (Wan et al., 2019); therefore, the DEPs involved in the biological processes leading to AL18A abortion might be essential for anthers fertility. Further analysis of these DEPs would significantly improve our understanding of anthers development.

**FIGURE 5:**
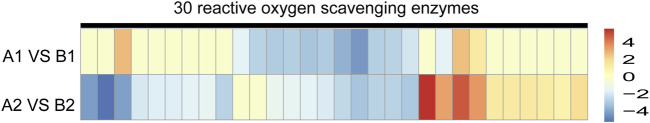
Expression analysis of DEPs in ROS scavenging enzymes of AL18A and AL18B at two anther development stages.

We further investigated the expression pattern of ROS scavenging enzymes in DEPs. The protein abundance of 30 ROS scavengers between AL18A and AL18B was significantly different (Figure 5; Supplementary Table S9). In AL18A anthers, 12 down-regulated and three up-regulated ROS scavenging enzymes were identified at the uni-nuclear stage; whereas, 18 down-regulated and ten up-regulated enzymes were observed at the bi-nucleated stage. These results were consistent with the transcriptome analysis that the redox homeostasis of AL18A is disrupted, which could explain our earlier discovery that AL18A’s tapetum PCD was delayed (Hao et al., 2021).

### Screening of Important Genes Related to Anthers Development

**FIGURE 6:**
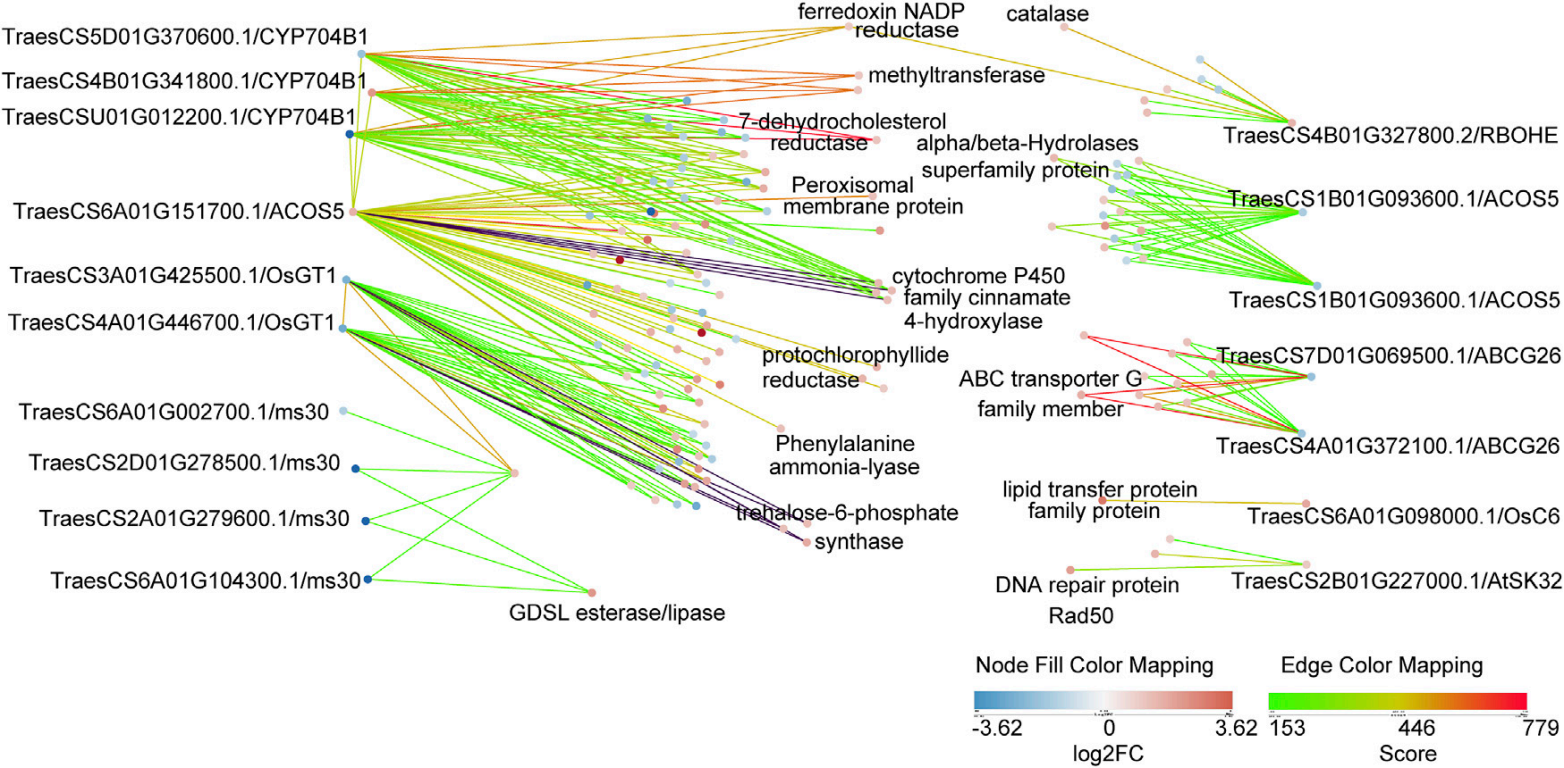
Protein interaction networks of 18 putative GMS orthologs genes in wheat. These 18 proteins were DEPs in bi-nucleate stage. The strength of protein interaction is represented by edge color, and the up-down relationship of DEPs is represented by node color. In addition, the putative GMS homologous genes were labeled in the outer part of the figure, and the gene annotations of some of their interacting proteins were labeled in the inner part of the figure.

Many GMS genes have been reported in rice, *Arabidopsis*, and maize, and most have conserved functions (Wan et al., 2019). However, few wheat GMS genes have been identified and functionally characterized so far. We performed the protein BLAST and the protein interaction analysis of DEPs, and subsequently detected 18 putative GMS orthologs in wheat and their 268 associated protein pairs interacting at the binuclear stage (Figure 6; Supplementary Tables S10, S11).

These results will be useful for the analysis of anther development mechanism. *RBOHE* in *Arabidopsis thaliana* encodes NADPH oxidase gene (Xie et al., 2014), TraesCS4B01G327800.2 is a putative homologue of *RBOHE* in wheat, which prediction interacts with catalase (TraesCS4B01G325800.1). *OsGT1* in rice encodes a glycosyltransferase (Moon et al., 2013), TraesCS4A01G446700.1 is a putative homologue of *OsGT1* in wheat, which prediction interacts with trehalose-6-phosphate synthase (TraesCS5B01G202200.1, TraesCS3A01G289300.1, TraesCS3D01G289100.1). *ABCG26* in *Arabidopsis thaliana* encodes a member of the ATP-binding cassette (ABC) transporter superfamily (Chang et al., 2016), TraesCS4A01G372100.1 is a putative homologue of *ABCG26* in wheat, which prediction interacts with ABC transporter G family member (TraesCS2A01G374100.1, TraesCS3A01G354500.1). *CYP704B1* in *Arabidopsis thaliana* encodes a long-chain fatty acid omega-hydroxylase, is essential for exine development (Dobritsa et al., 2009), TraesCS4B01G341800.1 is a putative homologue of *CYP704B1* in wheat, which prediction interacts with 7-dehydrocholesterol reductase (TraesCS3A01G488600.1), methyltransferase (TraesCS6A01G269700.1, TraesCS6B01G297000.1), ferredoxin NADP reductase (TraesCS6D01G012200.1). *ACOS5* in *Arabidopsis thaliana* encodes an enzyme that participates in a conserved and ancient biochemical pathway required for sporopollen in monomer biosynthesis (de Azevedo Souza et al., 2009), TraesCS6A01G151700.1 is a putative homologue of *ACOS5* in wheat, which prediction interacts with cytochrome P450 family cinnamate 4-hydroxylase (TraesCS3A01G136100.1, TraesCS3B01G154000.1, TraesCS3A01G342900.1, TraesCS3B01G375100.1), protochlorophyllide reductase (TraesCS1A01G171000.1, TraesCS1D01G168700.1, TraesCS2D01G563600.1), Phenylalanine ammonia-lyase (TraesCS6B01G258400.1), Peroxisomal membrane protein (TraesCS4A01G442900.1). The differential expression of these putative homologous of GMS proteins provided evidence for pollen abortion of AL18A, and these interesting interactions may be related to anthers development and need to be further explored.

**FIGURE 7:**
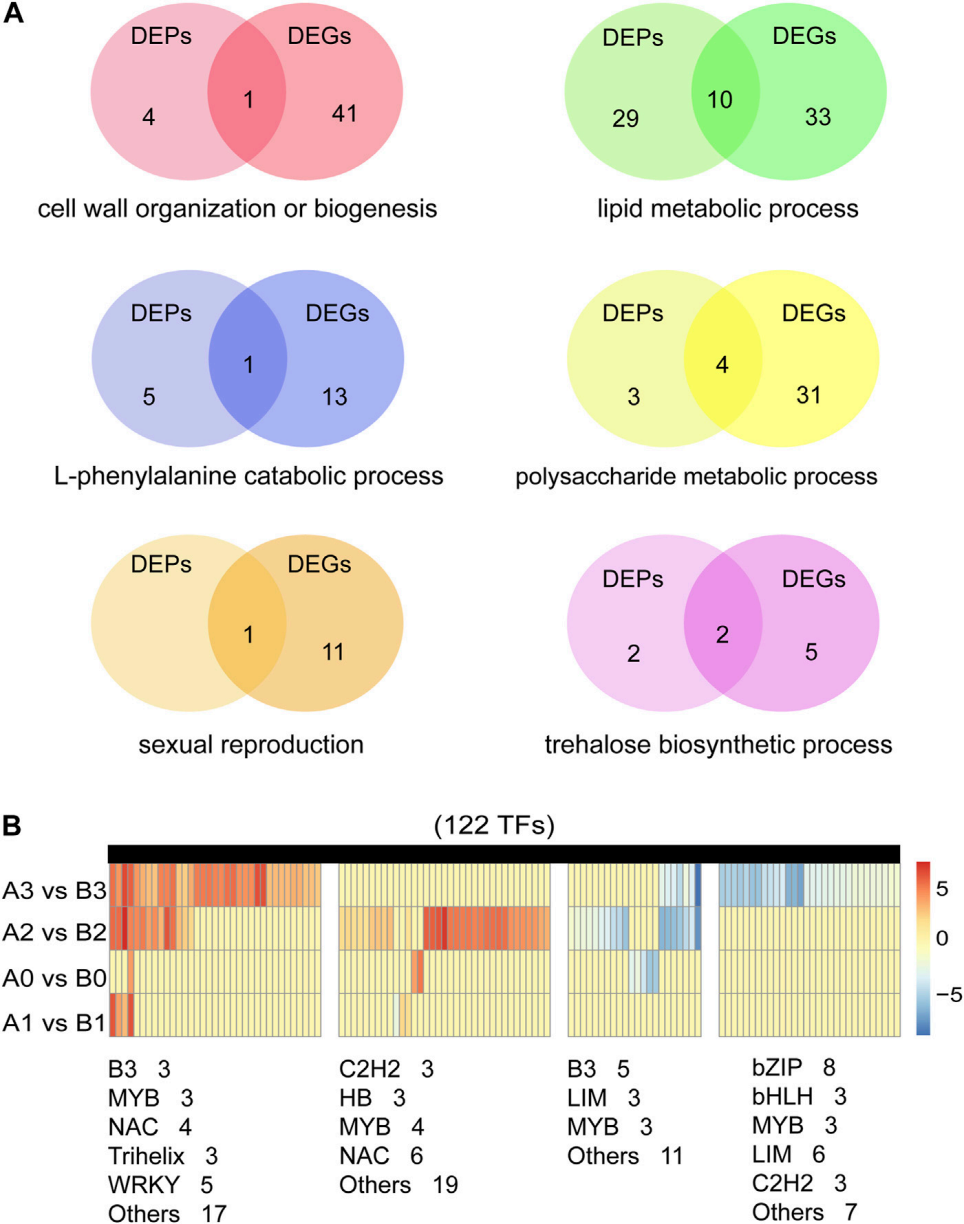
Screening of genes related to anthers development. **(A)** Identified of DEPs and DEGs in six important biological processes of anther development. **(B)** Expression analysis of 122 transcription factors in DEGs.

Previous studies have shown that biological processes such as redox homeostasis, lipid metabolism and polysaccharide metabolism are involved in tapetum PCD and pollen wall development (Wan et al., 2019). Similar results were obtained in our study. In addition, we found that trehalose biosynthesis process and sexual reproduction are also important for anthers development. We studied six important biological processes involved in anthers development (Figure 7A; Supplementary Tables S12, S13). A total of 46 cell wall organization or biogenesis related DEPs/DEGs, 24 encoding pectinesterase and nine encoding xyloglucan endotransglucosylase/hydrolase (Supplementary Table S14). 72 lipid metabolic process related DEPs/DEGs were detected, 14 encode 3-ketoacyl-CoA synthase, five encode GDSL esterase/lipase, 13 encode lipid transfer protein, seven encode Acyl-desaturase. Ten genes are both detected in DEPs and DEGs, five (TraesCS2A01G279600.1, TraesCS2D01G278500.1, TraesCS4B01G274700.1, TraesCS6A01G104300.1, TraesCS6B01G422900.1) of them are maize orthologs of ms30 (An et al., 2019), and PKSA/LAP6and PKSB/LAP5 in *Arabidopsis* (Kim et al., 2010). In the process of lipid metabolic process, we identified 15 DEGs that were putative homologue of the reported GMS (Supplementary Table S15). 19 L-phenylalanine metabolic process related DEPs/DEGs were detected, 14 encode phenylalanine ammonialyase, three encode 4-hydroxyphenylpyruvate dioxygenase and two encode glutathione S-transferase (Supplementary Table S16). 38 polysaccharide metabolic process related DEPs/DEGs were detected, two encode Alpha-1,4-glucan-protein synthase, 18 encode beta-amylase, four encode callose synthase. Eight (TraesCS2A01G122600.1, TraesCS3B01G333100.1, TraesCS4A01G166900.1, TraesCS7A01G146200.1, TraesCS7B01G048700.1, TraesCS7B01G243200.1, TraesCS7B01G480300.1, TraesCS7D01G554900.1) of them are rice orthologs of OsUAM3 (Sumiyoshi et al., 2015), and Cals 5 (Dong et al., 2005) and TPD1 (Jia et al., 2008) in *Arabidopsis* (Supplementary Table S17). In addition, we detected 12 DEPs/DEGs associated with sexual reproduction, 11 encode expansin protein (Supplementary Table S18). Nine DEPs/DEGs associated with trehalose biosynthetic process, seven encode trehalose-6-phosphate synthase, two (TraesCS2D01G168100.1, TraesCS5A01G190000.1) encode trehalose 6-phosphate phosphatase (Supplementary Table S19).

Anther development is one of the most critical events in the reproductive phase of plant development, involving hundreds of genes and proteins. Transcriptional factors (TFs) are the key connection points in regulatory networks associated with tapetal function and pollen development (Jiang et al., 2021). By comparing AL18A and AL18B, we analyzed the expression pattern of TFs in DEGs during the anther development (Figure 7B; Supplementary Table S20). As a result, we found 8, 7, 62, and 72 differentially expressed TFs at the tetrad, uni-nuclear, bi-nuclear, and tri-nuclear stages, respectively. The number of differentially expressed TFs increased as the anther developed from the uni-nuclear to the tri-nuclear stage, indicating that the anther abortion might have initiated at the uni-nuclear stage. In total, 122 differentially expressed TFs were identified, 40 of them were orthologs of ARF17 (Yang et al., 2013), CDM1 (Lu et al., 2014), DYT1 (Zhang et al., 2006), TDF1 (Zhu et al., 2008), and TGA9 (Murmu et al., 2010) from *Arabidopsis*, and Ocl4 (Vernoud et al., 2009) from maize (Supplementary Table S20). Although we have identified the GMS genes orthologs in wheat, whether these genes play similar roles in anthers development remains to be investigated.

## Discussion

### Redox Homeostasis is Important in AL18A Anther Development

Our research showed the abnormal electron transport chain at the uni-nuclear stage and significantly different expression patterns of ROS scavenging enzymes in AL18A compared to the AL18B (Figures 2, 5). Studies have shown that disturbance of the electron transport chain can affect redox homeostasis and the redox homeostasis in tapetum cells can regulate tapetum growth and PCD (Yu and Zhang, 2019). By interacting with *COX11*, a rice CMS gene *WA352* affects the removal of reactive oxygen species in tapetum cells and leads to the premature tapetum PCD, resulting in pollen abortion (Luo et al., 2013). As the CMS gene of AL18A is *orf279* and its function was annotated as an ATP synthase subunit 8 gene (Hao et al., 2021; Melonek et al., 2021). We speculate that the CMS gene may affect the homeostasis of reactive oxygen species by interfering with the electron transport chain, affecting the fertility of AL18A. More experiments are needed to confirm this hypothesis in the future.

### Genes Related to Anthers Development in Wheat

Studying genes by map-based cloning is time-consuming, and sometimes cloning of homologous genes is fast and effective. By analyzing the putative orthologs of GMS genes in *Arabidopsis* and rice, a putative regulatory network for anther and pollen development in maize was constructed (Wan et al., 2019; Jiang et al., 2021). In our study, DEGs/DEPs are involved in fatty acid biosynthesis process, lipid transport, L-phenylalanine metabolic process and polysaccharide metabolism in AL18A’s anthers development (Figure 7). The results are similar to previous studies in rice, *Arabidopsis thaliana* and maize (Wan et al., 2019). Meanwhile, we identified a number of GMS genes that are partially homologous to other species in wheat. The reverse genetics approach can be used to study these partially homologous GMS genes which are found in wheat.

### A Possible Working Model Related to Male Sterility in AL18A

**FIGURE 8:**
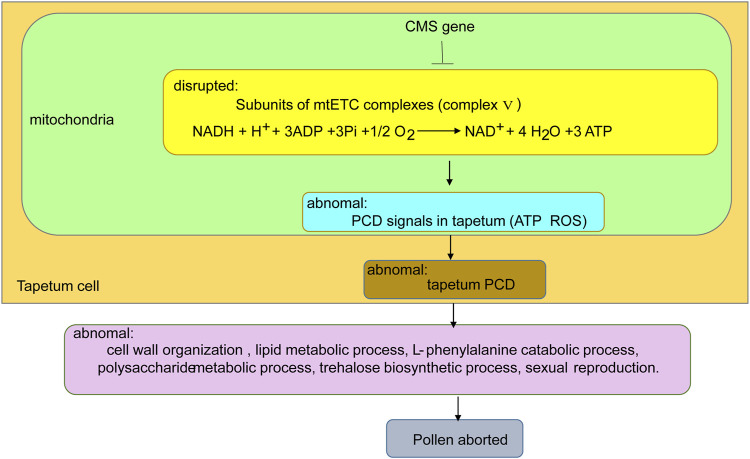
A proposed working model of pollen abortion in AL18A.

Based on previous reports and this study, we constructed a proposed working model of impaired anther development in the AL18A (Figure 8). The *orf279* of AL18A may changes ATP synthesis and ROS levels by affecting the mitochondrial electron transport chain of tapetum cells, which leads to delayed tapetum PCD (Hao et al., 2021). The aberrant tapetum PCD causes abnormality in cell wall organization, lipid metabolic process, L-phenylalanine metabolic process, polysaccharide metabolic process, trehalose biosynthetic process, and sexual reproduction. The aberration in the biological process causes pollen abortion in AL18A.

## Data Availability

The data presented in the study are deposited in the NCBI repository, accession number SAMN19979141–SAMN19979164.
