# Supplementary material for: Combined Transcriptome and Proteome Analysis of Anthers of AL-type Cytoplasmic Male Sterile Line and Its Maintainer Line Reveals New Insights into Mechanism of Male Sterility in Common Wheat
Source: Front Genet. 2021 Dec 17;12:762332. doi: 10.3389/fgene.2021.762332 (PMC8718765; doi:10.3389/fgene.2021.762332)
Supplement: Supplementary file 3 [file DataSheet2.ZIP › Figure S1 Venn map of differentially expressed transcripts of AL18A and AL18B at different anther development stages.pdf]

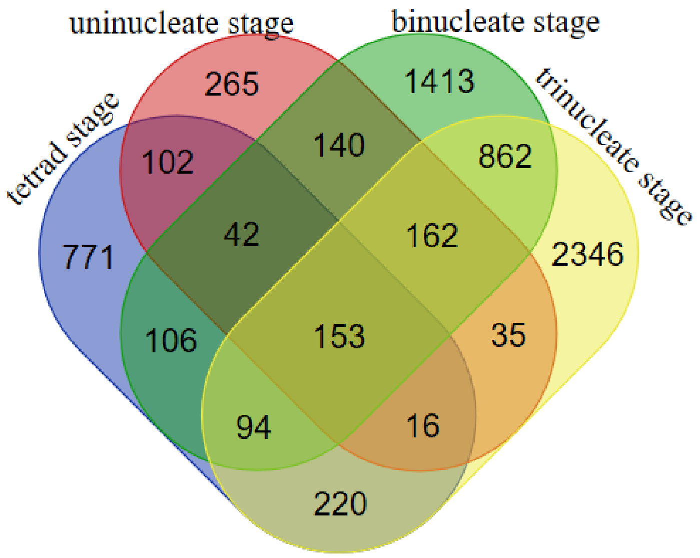

Figure S1 Venn map of differentially expressed transcripts of AL18A and AL18B at different anther development stages

The differentially expressed transcripts were: 1,505 in the tetrad stage; 916 in uninucleate stage; 2,973 in binucleate stage; 3,889 in trinucleate stage.
